# Clinical and Cost-Effectiveness of the “UCL Live Well With Parkinson's” Toolkit: A Randomised Controlled Trial

**DOI:** 10.1016/j.lanepe.2026.101762

**Published:** 2026-07-21

**Authors:** Anette Schrag, Kumud Kantilal, Tasmin Rookes, Benjamin Gardner, Gareth Ambler, Rachael Hunter, Nathan Davies, Catherine Atkinson, Patricia Schartau, Mariam Adeleke, Lina Gonzalez, Jiunn Wang, Maria Carmody, Kate Walters

**Affiliations:** a Department of Clinical and Movement Neurosciences, University College London, London, UK; b Research Department of Primary Care and Population Health, University College London, UK; c School of Psychology, University of Surrey, Guildford, UK; d Department of Statistical Science, University College London, London, UK; e Centre for Psychiatry and Mental Health, Wolfson Institute of Population Health, Queen Mary University of London, London, UK; f Homerton Healthcare NHS Foundation Trust, London, UK; g Centre for Primary Care, University Hospital Regensburg & University of Regensburg, Germany

**Keywords:** Parkinson's disease, Self-management, Randomised controlled trial, Digital, Intervention, Non-pharmacological

## Abstract

**Background:**

Self-management approaches in people with Parkinson's disease (PD) have potential to improve patient outcomes and reduce complications leading to hospital admissions. We aimed to evaluate the clinical and cost-effectiveness of the *UCL Live Well with Parkinson's toolkit,* a facilitated self-management intervention for people with PD.

**Methods:**

This two-arm randomised controlled trial in England (Trial Registration: ISRCTN92831552) recruited community-dwelling people with PD from NHS sites and self-referral. They were randomly assigned to the intervention or treatment as usual (TAU), and assessed at baseline, 6- and 12-month follow-up. The primary outcome was the PDQ-39 score, a PD-specific health-related quality of life measure, at 12-months with planned subgroup analyses. Secondary outcomes included non-motor and motor activities of daily living (MDS-UPDRS part I&II), utility values and QALYs derived from the EQ-5D-5L, and total health and social care costs over 12-months. The economic evaluation was based on cost-utility analysis using cost per QALY. All assessors were blinded to group allocation. Analysis was by intention to treat.

**Findings:**

166 participants were randomised to the intervention and 180 to TAU, with 12-month follow-up assessments available in 141 (84.9%) and 164 (91.1%), respectively. The primary endpoint (PDQ-39 score) was similar for patients in the intervention and TAU groups (−1.03; 95% CI (−3.03 to 0.97)). Subgroup analyses of PDQ-39 scores in underserved groups however favoured the intervention (−4.0; 95% CI (−6.8 to −1.1)). The combined MDS-UPDRS part I + II score was improved in the intervention compared to the TAU group (−2.61; 95% CI (−4.58 to −0.64)). QALYs were not different between groups (0.018; 95% CI (−0.006 to 0.042) but total health and social care costs over 12-months were lower in the intervention group compared to TAU (−£1282; 95% CI (−£2700 to −£118)), driven mainly by reduced unplanned hospital admissions. Adverse events were similar in both groups. The *Live Well with Parkinson's* intervention alongside TAU was 99% cost-effective compared to TAU at a decision threshold of £20,000 per QALY and 98% at £30,000.

**Interpretation:**

The *UCL Live Well with Parkinson's toolkit* did not significantly improve health-related quality of life scores overall but improved activities of daily living and reduced health-care costs in comparison to TAU, mainly through reduced unplanned hospital admissions.

**Funding:**

National Institute for Health and Care Research RP-PG-1016-20001.

**Research in context**

**Evidence before this study**

Parkinson's disease (PD) symptoms are usually managed through infrequent specialist appointments, which may be inadequate for the management of complex and evolving disease. Self-management interventions have been reported to improve health and quality of life outcomes for a range of other chronic diseases such as asthma and chronic back pain. To understand the evidence for self-management in people with Parkinson's, we searched MEDLINE, Embase, PsycINFO and Web of Science from inception to November 2020, and updated on 8th June 2026, for randomised controlled trials, using the terms “Parkinson”, combined with “randomised controlled trial” and “self-management”. All studies identified were checked against the established definition of self-management. Self-management interventions were heterogeneous in approach and outcomes. Risk of bias was moderate to high for most studies, with low study quality and small sample sizes. Nevertheless, benefits of self-management interventions have been reported in small-scale studies for a variety of outcome measures, such as quality of life, fatigue, wellbeing, self-care management, medication adherence and functioning. No studies have evaluated the cost effectiveness of self-management interventions in Parkinson's disease.

**Added value of this study**

This is the first large, randomised controlled trial of a facilitated self-management intervention in People with Parkinson's in England. The trial demonstrated that a facilitated self-management intervention in people with Parkinson's did not improve the primary outcome (health-related quality of life) overall. However, it improved functional and mental health outcomes, was cost-effective, and improved health-related quality of life in underserved groups. The intervention was also associated with cost-savings, particularly through reduction in unplanned hospital admissions.

**Implications of all the available evidence**

Although overall health-related quality of life scores did not improve, facilitated self-management interventions in PD such as the *UCL Live Well with Parkinson's* intervention can reduce costs to health-care systems and improve functional outcomes relating to motor and non-motor features. Populations in rural areas, where access to specialist services is more limited, may be most likely to benefit from such interventions.

## Introduction

Traditional approaches to managing Parkinson's disease (PD) in the UK involve scheduled outpatient appointments with specialists in addition to management by primary care physicians and, where available, support through PD specialist nurses. However, this model is increasingly recognised to be inadequate for the management of a complex disorder with multiple possible symptoms^1^ may require specialist input but can potentially be managed by other healthcare providers or patients themselves with the appropriate knowledge and information. Crucially, Parkinson's disease is a highly heterogeneous condition, for which no standard approach exists that fits all people with Parkinson's disease. This requires a personalised approach, not only to fit with the clinical symptoms but also personal circumstances, demographics, comorbidities, priorities and social and personal characteristics. Timely identification of newly arising symptoms or complications has the potential to prevent deterioration and unplanned hospital admissions and improve patient outcomes, including quality of life, functioning, and wellbeing. Enabling patients and their families to have greater self-efficacy to control their own symptoms and disease management can potentially lead to earlier recognition and prevention of deterioration. Hence, there is an increasing shift towards enabling self-management in People with Parkinson's. Limitations to this approach include managing the complexity of this progressive disease and vast sources of available information both analogue and digital which can be overwhelming for people with Parkinson's to tailor and requires knowledge of the disease symptoms and treatments.

We therefore set out to develop an intervention to provide education and information to facilitate self-management of symptoms arising in the context of Parkinson's disease, supported by trained facilitators for personalised goal setting, self-monitoring, and to guide people with Parkinson's and their carers in use of the tool, between their regular appointments with specialists. The development of the *UCL Live Well with Parkinson's* toolkit utilised co-design methods with People with Parkinson's, carers of people with Parkinson's, a range of healthcare professionals (HCPs) and researchers,^2^ as well as qualitative interviews with people with Parkinson's, carers and HCPs working with people with Parkinson's,^3^, ^4^, ^5^ reviewing the existing relevant literature,^6^, ^7^, ^8^ and conducting a feasibility study,^9^ which showed that the trial would be feasible and acceptable.

## Methods

This was a single-blinded, superiority randomised controlled trial (RCT) with two parallel arms, which aimed to compare the clinical and cost-effectiveness of the ‘*UCL Live Well with Parkinson's’* Facilitated Self-Management Toolkit (intervention) with Treatment as usual (TAU). The RCT was prospectively registered and includes the statistical analysis plans (ISRCTN92831552). The protocol has been published.^10^ The findings of this RCT are reported in accordance with CONSORT recommendations. We also undertook a parallel mixed methods process evaluation which will be reported separately.

### Eligibility criteria

Participants were eligible if they were community-dwelling adults aged 18 and above with a confirmed diagnosis of Parkinson's disease (defined using UK Brain Bank Criteria^11^), including those with dementia diagnosed at least one year after their Parkinson's disease diagnosis. Participants needed to be able to engage in the intervention and study assessments independently or with the support of a carer or family member.

Participants were excluded if they had a clinical diagnosis of Atypical Parkinsonism; were inpatients or living in a care home; had a telephone Montreal Cognitive Assessment (MoCA)^12^ score <11 or lacked the capacity to take part; were unable to engage in the intervention due to visual impairment or language barriers (and had no carer or family member to support them to engage); had a life expectancy <6-months; or were participating in another clinical study likely to impact or interfere with the *UCL Live Well with Parkinson's* intervention.

### Recruitment methods and setting

Recruitment was conducted through secondary and primary care in England between 19th January 2022 and 11th July 2023. Sites were selected to represent a range of settings, including teaching hospitals, district general hospitals and primary care across inner city, suburban and rural locations. Given the known under-recruitment of clinical trials from rural, less affluent areas and diverse ethnic groups, we made particular efforts to recruit from such areas and populations, particularly inviting GP practices in areas ranked 1–3 on the Index of Multiple Deprivation (IMD) deciles (1 representing the most socioeconomically deprived 10% of areas in England). The study team collaborated with Parkinson's UK to further support recruitment through their trial finder (www.parkinsons.org.uk).

### Randomisation and masking

Participants were randomised 1:1 to receive the *UCL Live Well with Parkinson's* intervention or TAU. Minimisation was used to perform individual randomisation based on site. Randomisation was carried out using a ‘Sealed Envelope’ remote computerised web-based application. Outcome assessors, statisticians, site investigators, the Chief Investigators, the Trial Manager and Trial Management Group members were all blinded to participant allocation.

### Study intervention

The intervention was the manualised *UCL Live Well with Parkinson's* intervention in addition to TAU, supported by a trained facilitator with up to six sessions over 6 months.

#### The UCL Live Well with Parkinson's Toolkit

The Toolkit incorporated evidence from effective health promotion interventions and behaviour change techniques (BCTs) drawn from the COM-B model.^13^ An asset-based approach underpinned the overall intervention, focussing on maintaining independence, health, and current activities. The Toolkit was co-designed with people with Parkinson's, carers, HCPs and Parkinson's disease experts and was made available in paper and online formats. Both versions could be shared with participants' carers and HCPs, either as a whole toolkit or selected sections.

The Toolkit consists of 64 information sections on Parkinson's disease, symptoms, therapies/treatments, optimising wellbeing, and practical advice. It also contains personalised sections completed by the participant titled as follows: 1) About Me (including information on their contacts, support and planning future care); 2) My Health (including information on their health conditions, medication, treatments, and research involvement); 3) Symptom Review (including a list of symptoms they experience and the severity of them); 4) My Wellbeing (to identify health behaviours they would like to maintain or improve); 5) My Tracker (to track and self-monitor medications, activities, and symptoms allowing participants and specialists to identify patterns); 6) Appointments/calendar (to allow participants to store all their healthcare appointments in one place); and 7) To-do lists/Notes. Additional information about the Toolkit development and contents, and a TIDieR checklist is available elsewhere.^2^^,^^10^

#### Intervention supporter sessions

Participants and, if participants wished, their carers, received up to six sessions over 6-months with a ‘supporter’. Supporters were trained professionals with a background in health or social care (e.g., psychology, occupational therapy, nursing, social care), or third-sector organisations (e.g., care navigation, social prescribing), with some experience working/caring for people with Parkinson's or other complex long-term conditions. Supporter training, supervision, and session procedures are published elsewhere.^9^^,^^10^

The sessions aimed to encourage participants to self-manage their condition using the *UCL Live well with Parkinson's* Toolkit with all its components, including the information, self-monitoring and well-being sections, personalised to the individual, and using behaviour change techniques to support goal-setting, action planning and problem-solving, tailored to their circumstances. These sessions were conducted online via video call, by telephone, or face-to-face depending on participants' access to technology and preference.

### Treatment as usual (TAU) arm

The TAU arm continued to receive their usual care from existing sources (GP, Parkinson's disease specialist service ± NHS Parkinson's Disease Nurse Specialists (PDNS), community therapy teams). Usual Care in the current NHS is delivered by primary care in most instances with secondary care (neurology or geriatrics) outpatient consultations every 6- to 12-months, and with a PDNS, where available, who can provide information, reviews, and a telephone service for queries between appointments. Referrals to other specialities and therapists are made as appropriate. There is however considerable heterogeneity in the availability of specialist services and their organisation.

### Outcomes

Clinical outcomes were measured at baseline, 6-months and 12-months by a researcher blind to intervention status. Assessments were completed face-to-face at a clinic or the participant's home or remotely by video or telephone. Assessments were divided into two sessions with some assessments completed by the participant alone and some with a researcher present. Participants received a £20 voucher for completing the baseline assessments and £10 for each follow-up completed. Data were kept confidentially at sites or in the UCL Data Safe Haven, anonymised and entered into a ‘Sealed Enveloped’ secure web-based database, developed for the trial. A monitoring plan was followed to ensure data quality.

The two key outcomes were the PDQ-39, a PD-specific health-related quality of life measure, and the MDS-UPDRS experiences of daily living parts (part I&II), for which the study power calculation was the same. Following the feasibility study^9^ we chose the PDQ-39 as our primary outcome as available data suggested it may be more sensitive to change with the intervention, but we considered the MDS-UPDRS to be a similarly important outcome. Secondary outcomes were non-motor and motor experiences of daily living, motor examination findings and motor complications (MDS-UPDRS parts I & II, part III, and part IV); non-motor symptoms (Non-Motor Symptoms Rating Scale (MDS-NMS); psychological wellbeing (General Health Questionnaire [GHQ-12]); health related quality of life and a visual analogue scale (VAS) of current health status (EQ-5D-5L); health service use in the preceding 3-months (Client Service Receipt Inventory-shortened (CSRI), adapted for Parkinson's disease); and capability among older people (Investigating Choice Experiments Capability measure for Older people (ICECAP-O). Utility tariffs for the EQ-5D-5L were calculated in line with NICE guidance.^14^^,^^15^ At baseline, demographics were collected included age, gender, ethnicity, marital status, education level, deprivation indices, employment status, sexual orientation, living situation, living location, other long-term conditions, and the telephone MoCA to assess cognitive impairment. For process evaluation purposes, several additional measures were collected, analyses of which will be reported separately.^10^

### Sample size

To detect a 4.7-point difference in our primary outcome PDQ-39^16^ with 90% power and 5% significance using a baseline-adjusted (ANCOVA) analysis and assuming a Standard Deviation of 19.8 and a correlation between baseline and follow-up measurements of 0.8,^17^^,^^18^ we required 135 participants per arm. Allowing for 20% attrition at 12-months in each arm we required 338 participants in total. The Standard Deviation of 19.8 was derived from the PDQ-39-SI document included in the tables of our published protocol.^10^ The assumed correlation of 0.8 between baseline and follow-up measurements was based on results from the feasibility study for the secondary outcome MDS-UPRDS part I&II,^9^^,^^16^ which was initially planned as the primary outcome measure.

### Statistical methods

A statistical analysis plan was agreed and published with the protocol.^10^ Analyses were conducted in Stata 18.^19^ The primary analysis used a three-level linear mixed model to compare PDQ-39 scores at 12-months between treatment groups, adjusting for baseline PDQ-39, age and IMD as fixed effects, and participant and site as random effects. All analyses were performed on an intention-to-treat basis, and all modelling assumptions were checked. Secondary outcomes were compared using similar methods to the primary outcome.

We performed a Complier Average Causal Effect (CACE) analysis using 2-stage least squares to estimate the effect of the intervention on PDQ-39 among those patients who would comply with the intervention.^20^

The PDQ-39 has 39 items in 8 domains, and individual missing items were imputed using the domain average for that patient if there were 4 or less missing items. As a sensitivity analysis, we used multiple imputation (with chained equations)^21^ to impute missing values of the PDQ-39 for each arm separately, based on baseline PDQ-39, age and indices. We also used reference-based imputation to impute under various ‘missing not at random’ (MNAR) assumptions.^22^ As the results were similar to the main analysis, we only report the main analysis findings.

We also undertook pre-specified sub-group analyses, using interaction terms, to investigate effectiveness by those belonging to an underserved population (leaving education before or after age 16 years, white vs non-white ethnicity, deprivation index 1–3 or 4–10, and living in rural areas or not) and by Parkinson's disease severity (measured using the MDS-UPDRS part I&II: 0–22 mild, 23–50 moderate and 51+ severe disease severity^23^). We had originally planned to use Hoehn and Yahr staging for the severity sub-group analysis; however, we were unable to rate disease severity in 84/346 (24.3%) of participants. We therefore used the MDS-UPDRS part I&II as a measure of functional severity rating, for which we had data for most participants (342/346, 98.8%).

#### Economic evaluation

A health economic analysis plan was agreed prior to analysis.^10^ We conducted a cost-utility analysis calculating the incremental costs per quality-adjusted life years (QALYs) associated with the intervention compared with TAU, from both health and social care and societal perspectives.

Health care resource use components were costed using the Personal Social Services Research Unit (PSSRU) unit costs of health and social care and NHS reference costs 2022/2023^24^^,^^25^ (Supplementary Table S14).

The cost of the intervention included intervention delivery; consumables for the paper manual or website hosting and maintenance for the digital Toolkit and expenses related to the facilitators in terms of training, staff time, line management, and supervision. The average intervention cost was calculated by dividing the total cost of the intervention by the number of participants in the treatment group.

EQ-5D-5L responses were converted into utility scores using UK population valuation weights.^26^^,^^27^ Utility from 0 (death) to 1 (full health), with negative values representing health states considered worse than death. Participant-specific utility profiles were generated by linear interpolation between utility values recorded at each measurement point.

QALYs for the intervention compared to the control group were calculated using linear regression adjusting for baseline, socio-economic fixed effects and site as a random effect. To account for the correlation between costs and QALY, we calculated mean incremental costs and QALYs using seemingly unrelated regression (SUR) adjusting for baseline, age, IMD, site, and marital status.^28^ Bootstrapping was used to calculate 95% confidence intervals, cost-effectiveness acceptability curves (CEAC), and cost-effectiveness planes (CEP). Results are reported from a health and personal social services and wider societal perspective. A sensitivity analysis tested the impact on mean incremental costs of varying the intervention cost between a maximum (£417 per participant) and minimum value (£237 per participant). There was no discounting as the time horizon does not go beyond 12-months.

### Ethics and patient and public involvement (PPI)

A study-specific PPI Advisory Panel of eight people with lived experience of Parkinson's or of caring for someone with Parkinson's acted as an advisory group for all stages of this RCT. The Advisory Panel lead (MC) was a member of the RCT management group. The study was approved on 18 October 2021 by London—Harrow Research Ethics Committee (REC) and Health research Authority (HRA) (reference 21/LO/0562). Informed consent was obtained from all participants prior to data collection.

### Role of the funding source

The funder had no role in study design, data collection, data analysis, data interpretation, or writing of the report or the decision to submit.

## Results

**Fig. 1:**
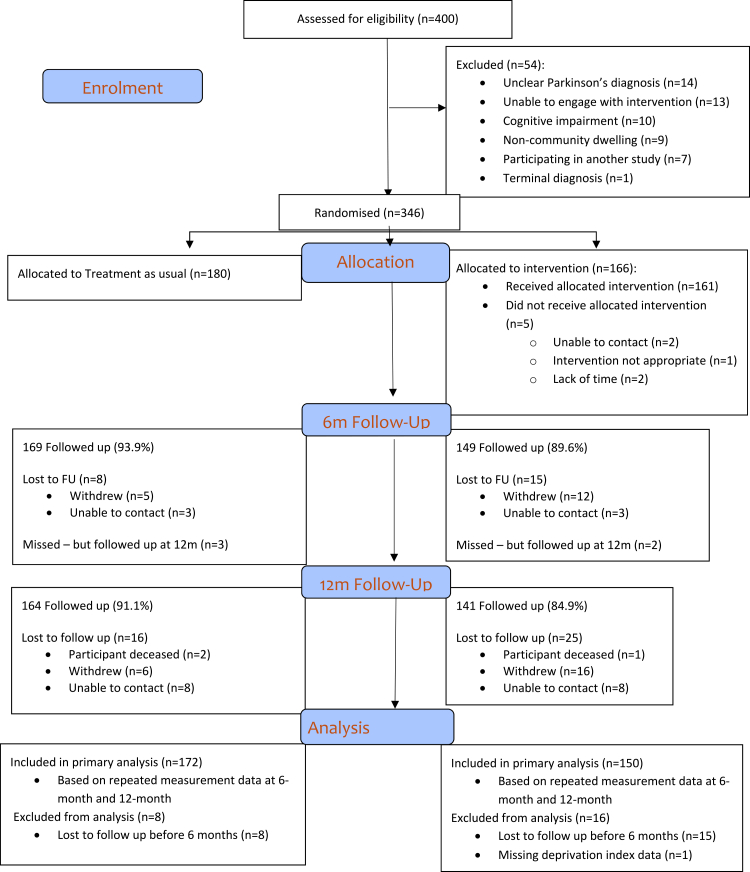
Consort diagram and recruitment and retention.

We randomised 346 participants out of 400 individuals assessed for eligibility to either the *UCL Live Well with Parkinson's* intervention (N = 166) or TAU (N = 180) between January 2022 and July 2023 (Fig. 1). Ninety three percent of participants completed at least one follow-up assessment.

Participants had a mean age of 69.1 (SD 9.3; range 36–89) years and 54% (187/346) were male (Table 1). Most participants were white (321/346, 92.8%), married or in a civil partnership (245/346, 70.8%) and living with their spouse/partner (224/346, 64.7%). Most were retired (256/346, 74.0%) with 16.8% (58/346) in full-or part-time employment. About one in five participants (79/345) lived in the most socioeconomically deprived neighbourhoods in England (IMD decile 1–3) and 150/345 (43.5%) were living in neighbourhoods in IMD decile 7–10, with a mean IMD decile of 5.9 (SD 2.7). Whilst most were living in main cities (171/346, 49%) or towns (114/346, 33%), 18% (61/346) lived in rural areas. Mean baseline MDS-UPDRS part I score was 11.2 (6.5) and part II score was 12.1 (7.4), with 181 (52.5%) having mild, 155 (44.9%) moderate and 9 (2.6%) severe PD, as measured with the MDS-UPDRS part I&II. Demographic and clinical characteristics were similar between both arms.

**Table 1:** Baseline demographic characteristics (n (%)).

	UCL Live Well with Parkinson's (N = 166)	TAU (N = 180)	Total (N = 346)
Gender			
Male	92 (55.4%)	95 (52.8%)	187 (54.0%)
Female	74 (44.6%)	85 (47.2%)	159 (46.0%)
Age–mean (SD)	68.8 (9.0)	69.5 (9.6)	69.1 (9.3)
Ethnicity			
White	157 (94.6%)	164 (91.1%)	321 (92.8%)
Mixed/Multiple ethnic groups	0 (0%)	1 (0.6%)	1 (0.3%)
Asian/Asian British	6 (3.6%)	10 (5.6%)	16 (4.6%)
Black/African/Black	2 (1.2%)	5 (2.8%)	7 (2.0%)
Caribbean/Black British	0 (0%)	0 (0%)	0 (0%)
Other ethnic group	1 (0.6%)	0 (0%)	1 (0.3%)
Marital Status			
Single/Unmarried	11 (6.6%)	19 (10.6%)	30 (8.7%)
Co-habiting	5 (3.0%)	9 (5.0%)	14 (4.0%)
Separated	3 (1.8%)	3 (1.7%)	6 (1.7%)
Married/civil partnership	128 (77.1%)	117 (65.0%)	245 (70.8%)
Widowed	11 (6.6%)	24 (13.3%)	35 (10.1%)
Divorced	8 (4.8%)	8 (4.4%)	16 (4.6%)
Residence			
Major conurbation	20 (12.0%)	23 (12.8%)	43 (12.4%)
City (city with abundant services)	55 (33.1%)	73 (40.6%)	128 (37.0%)
Town	57 (34.3%)	57 (31.7%)	114 (32.9%)
Village	29 (17.5%)	19 (10.6%)	48 (13.9%)
Hamlet & isolated dwelling	5 (3.0%)	8 (4.4%)	13 (3.8%)
IMD decile (N = 345)			
1–3 (most deprived)	32 (19.4%)	47 (26.1%)	79 (22.9%)
4–6	63 (38.2%)	53 (29.4%)	116 (33.6%)
7–10 (least deprived)	70 (42.4%)	80 (44.4%)	150 (43.5%)
Mean (SD)	6.0 (2.7)	5.8 (2.8)	5.9 (2.7)
Living Arrangements			
Lives with spouse/life-partner	107 (64.5%)	117 (65.0%)	224 (64.7%)
Lives with family	36 (21.7%)	24 (13.3%)	60 (17.3%)
Live with full-time care giver/support	1 (0.6%)	1 (0.6%)	2 (0.6%)
Lives alone	22 (13.3%)	35 (19.4%)	57 (16.5%)
Lives with Other	0 (0%)	3 (1.7%)	3 (0.9%)
Employment status			
Full-time employed	15 (9.0%)	15 (8.3%)	30 (8.7%)
Part-time employed	16 (9.6%)	12 (6.7%)	28 (8.1%)
Unable to work due to illness	9 (5.4%)	9 (5.0%)	18 (5.2%)
Self-employed	6 (3.6%)	5 (2.8%)	11 (3.2%)
Retired	119 (71.7%)	137 (76.1%)	256 (74.0%)
Unemployed	1 (0.6%)	2 (1.1%)	3 (0.09%)
Age left education			
<16	17 (10.2%)	24 (13.3%)	41 (11.8%)
16–17	48 (28.9%)	39 (21.7%)	87 (25.1%)
18–25	90 (54.2%)	104 (57.8%)	194 (56.1%)
26≤	11 (6.6%)	13 (7.2%)	24 (6.9%)
Hoehn and Yahr stage			
1: Unilateral involvement only	45 (27.1%)	50 (27.8%)	95 (27.5%)
2: Bilateral involvement without impairment of balance	32 (19.3%)	42 (23.3%)	74 (21.4%)
3: Mild to moderate involvement	36 (21.7%)	36 (20.0%)	72 (20.8%)
4: Severe disability; still able to walk or stand unassisted	11 (6.6%)	9 (5.0%)	20 (5.8%)
5: Wheelchair bound or bedridden unless aided	1 (0.6%)	0 (0%)	1 (0.3%)
Unable to rate	41 (24.7%)	43 (23.9%)	84 (24.3%)
Disease severity (MDS-UPDRS)			
Part I			
Mild (0–10)	85 (51.5%)	92 (51.1%)	177 (51.3%)
Moderate (11–21)	66 (40.0%)	75 (41.7%)	141 (40.9%)
Severe (22+)	14 (8.5%)	13 (7.2%)	27 (7.8%)
Part II			
Mild (0–12)	99 (60.0%)	108 (60.0%)	207 (60.0%)
Moderate (13–29)	60 (36.4%)	66 (36.7%)	126 (36.5%)
Severe (30+)	6 (3.6%)	6 (3.3%)	12 (3.5%)
Part I and II			
Mild (0–22)	91 (55.2%)	90 (50.0%)	181 (52.5%)
Moderate (23–50)	70 (42.4%)	85 (47.2%)	155 (44.9)
Severe (51+)	4 (2.4%)	5 (2.8%)	9 (2.6%)

IMD = Index of Multiple Deprivation.

Most participants in the intervention arm (125, 75.8%) elected to use the online version, 39 (23.6%) used the paper version, and one (0.6%) participant used both the online and paper toolkit. Their characteristics are shown in Supplementary Table S10. Intervention sessions were well attended (mean attendance 5.25 appointments, SD 0.71), with 138/166 (83%) attending the prespecified minimum clinically effective dose of at least four of the six planned appointments. 762 intervention sessions were conducted remotely either via phone or video conferencing, and only 7 in person, following participant preference.

Compared to baseline, the primary outcome, the mean PDQ-39 scores at 12-months marginally deteriorated in the TAU group and improved slightly in the intervention group (Table 2). There was no statistically significant difference in mean PDQ-39 scores in the intervention group compared to the TAU group (−1.03; 95% CI (−3.03 to 0.97); *p* = 0.31). Pre-defined subgroup analysis of underserved groups suggests that the effect of the intervention varied by group, with significant improvement in PDQ-39 score at 12-months in underserved groups (left school before 16, non-white ethnicity, IMD score 1–3, or living in a rural area) (−4.0; 95% CI (−6.8 to −1.1; group interaction *p* = 0.01)). Individual subgroup analysis for each of these groups was significant only for those living in a rural area who had a greater improvement than those in other areas (Supplementary Table S2). Other subgroup analyses by disease severity (MDS-UPDRS part I&II), session attendance (CACE), and toolkit choice did not yield significant effects (Supplementary Table S3, S9 and S11).

**Table 2:** PDQ-39 scores at Baseline, 6-months and 12-months.

PDQ-39	TAU		Intervention		Adjusted mean difference (95% CI)	p-value
	N	Mean (SD)	n	Mean (SD)		
Baseline	180	22.98 (14.01)	166	22.29 (14.11)		
6-months	168	22.41 (14.73)	149	21.00 (14.16)	−1.12 (−3.09 to 0.85)	0.27
12-months	164	23.48 (14.82)	141	21.86 (14.92)	−1.03 (−3.03 to 0.97)	0.31

There was a significant difference in the mean combined MDS-UPDRS part I&II score at 6-months (−2.19; 95% CI (−3.95 to −0.43)) and at 12-months (−2.61; 95% CI (−4.58 to −0.64)) in favour of the intervention group, indicating improved motor and non-motor experiences of daily living (Table 3). Mean differences in the intervention group showed larger improvements in non-motor symptoms (the MDS-NMS) at 6-months (−13.22; 95% CI (−23.63 to −2.81)), with a significant difference in the NMS subdomains for depression at 6-months (−3.38; 95% CI (−5.26 to −1.49)) and 12-months (−2.44; 95% CI (−4.55 to −0.33)) and for psychosis at 12-months (−0.72; 95% CI (−1.39 to −0.06)) (Supplementary Table S4). There was also a significant difference in favour of the intervention group in the GHQ-12 scores at 6-months (−0.87; 95% CI (−1.71 to −0.03)). There was no difference in the EQ-5D-5L utility index score at 6- or 12-months, but the participants’ evaluation of their health on the EQ-5D-5L VAS at 6-months was higher in the intervention group than the TAU group, indicating better self-reported health status in that group (3.87; 95% CI (0.32–7.41)).

**Table 3:** Secondary outcome scores at Baseline, 6-months and 12-months.

Outcome	TAU		Intervention		Adjusted mean difference (95% CI)
	n	Mean (SD)	n	Mean (SD)	
MDS-UPDRS parts I & II score					
Baseline	180	23.38 (11.97)	165	23.25 (12.60)	
6-month	168	24.74 (13.41)	149	22.44 (13.17)	**−2.19 (−3.95 to −0.43)**
12-month	162	27.30 (13.99)	141	24.43 (13.78)	**−2.61 (−4.58 to −0.64)**
MDS-UPDRS part I score					
Baseline	180	11.38 (6.30)	165	11.04 (6.64)	
6-month	168	12.10 (7.36)	149	10.76 (6.63)	−1.03 (−2.08 to 0.02)
12-month	162	13.14 (7.49)	141	11.61 (6.80)	−1.08 (−2.26 to 0.11)
MDS-UPDRS part II score					
Baseline	180	12.00 (7.18)	165	12.20 (7.60)	
6-month	169	12.69 (7.66)	149	11.68 (8.20)	**−1.23 (−2.30 to −0.17)**
12-month	162	14.16 (8.20)	141	12.82 (8.46)	**−1.59 (−2.76 to −0.42)**
MDS-UPDRS part III score^a^					
Baseline	72	27.70 (14.63)	69	28.24 (15.24)	
6-month	57	30.64 (15.00)	64	29.14 (14.08)	−2.37 (−6.72 to 1.98)
12-month	61	38.83 (16.66)	57	37.71 (15.47)	3.10 (−1.91 to 8.12)
MDS-UPDRS part IV score					
Baseline	172	4.20 (4.18)	164	4.14 (4.24)	
6-month	157	3.90 (3.99)	146	3.92 (4.13)	0.08 (−0.63 to 0.78)
12-month	154	3.73 (4.02)	133	3.76 (4.36)	0.02 (−0.82 to 0.86)
MDS-NMS score					
Baseline	172	95.59 (71.23)	156	96.99 (81.83)	
6-month	141	93.35 (70.84)	126	87.96 (68.68)	**−13.22 (−23.63 to −2.81)**
12-month	130	96.32 (62.15)	108	87.39 (65.34)	−9.48 (−21.19 to 2.24)
GHQ-12 score					
Baseline	179	13.50 (5.71)	164	13.15 (5.37)	
6-month	169	12.95 (5.02)	150	12.09 (5.25)	**−0.87 (−1.71 to −0.03)**
12-month	162	13.83 (5.76)	142	13.04 (5.27)	−0.71 (−1.78 to 0.36)
EQ-5D-5L VAS					
Baseline	180	67.18 (19.70)	166	67.98 (18.58)	
6-month	170	63.57 (20.60)	150	67.40 (19.57)	**3.87 (0.32–7.41)**
12-month	164	63.85 (19.68)	144	65.42 (19.60)	1.29 (−2.54 to 5.11)
EQ-5D-5L mapped to the EQ-5D-3L					0.018 (−0.006 to 0.042)^b^ 0.015 (−0.012 to 0.041)^c^
Baseline	180	0.630 (0.232)	166	0.626 (0.197)	
6-month	170	0.622 (0.237)	150	0.628 (0.200)	
12-month	165	0.590 (0.256)	144	0.615 (0.222)	
12-months QALYs	162	0.617 (0.215)	140	0.623 (0.190)	

a Missing data points for 3.3–rigidity (5 items) and 3.12–postural stability for most participants due to remote assessment.

b Controlling for baseline.

c Accounting for correlation with costs.

The mean total cost per participant of the *UCL Live Well with Parkinson's* intervention was £284 (SD £66). The cost of consumables per participant is £62, the cost of training, supervision, and line management per participant is £41, and the cost of the actual delivery of the intervention is £189 per participant (Supplementary Table S6). When accounting for the cost of the intervention, the total health and social care costs per person over 12-months were £1282 lower in the *UCL Live Well with Parkinson's* group than in the TAU group (Table 4). The greatest contribution to difference in costs was seen in unplanned hospital admissions, planned hospital admissions and home adaptations (Supplementary Table S7). The incidence rate ratio revealed that the intervention reduces hospital admissions by roughly 71% (0.29 IRR, 95% CI (0.076–1.094, p = 0.068)), in comparison to the control group, although this finding was not statistically significant. From a health and personal social services perspective, the *UCL Live Well with Parkinson's* intervention cost less than TAU (−£1282; 95 CI% (−£2700 to −£118)). From a wider societal perspective, the adjusted mean difference accounting for correlation with QALYs was −£2682 (95% CI −£9687 to £2764) favouring the intervention, however this was not statistically significant.

**Table 4:** Cost components and total costs.

	Intervention		TAU	
	n	Mean (SD)	n	Mean (SD)
Community health services				
Baseline	166	210 (442)	180	207 (420)
6-months	149	142 (232)	167	149 (207)
12-months	138	166 (294)	159	117 (181)
Total at 12-months	138	306 (429)	157	267 (290)
Emergency Services				
Baseline	166	23 (99)	180	20 (79)
6-months	149	24 (105)	167	26 (116)
12-months	138	14 (62)	159	39 (122)
Total at 12-months	138	36 (118)	157	67 (206)
Hospital stays (unplanned)				
Baseline	166	105 (688)	180	136 (966)
6-months	149	117 (787)	167	80 (553)
12-months	138	23 (212)	159	561 (3979)
Total at 12-months	138	137 (832)	157	654 (4072)
Hospital stays (planned)				
Baseline	166	0 (0)	180	243 (1668)
6-months	149	126 (882)	167	300 (2731)
12-months	138	91 (751)	159	561 (3979)
Total at 12-months	138	227 (1173)	157	438 (3177)
Specialist outpatient visits				
Baseline	166	658 (1045)	180	587 (1014)
6-months	149	550 (904)	167	605 (1051)
12-months	138	615	159	652 (852)
Total at 12-months	138	1151 (1326)	157	1255 (1500)
Local transport (state-funded)				
Baseline	166	1 (7)	180	0.17 (2)
6-months	149	0.41 (5)	167	1 (9)
12-months	138	0 (0)	159	0.38 (5)
Total at 12-months	138	0.44 (5)	157	1 (11)
Home adaptations (state-funded)				
Baseline	166	685 (2421)	180	909 (3038)
6-months	149	700 (2857)	167	1011 (2941)
12-months	138	439 (1897)	159	748 (2547)
Total at 12-months	138	1056 (3088)	157	1707 (3724)
Medication cost				
Baseline	166	131 (142)	180	143 (219)
6-months	149	77 (75)	167	74 (77)
12-months	138	110 (266)	159	129 (202)
Total at 12-months	138	185 (283)	157	203 (237)
Personal care and help at home (state funded)				
Baseline	166	18 (104)	180	143 (219)
6-months	149	14 (173)	167	74 (77)
12-months	138	112 (934)	159	129 (202)
Total at 12-months	138	127 (949)	157	203 (237)
Total health and social care resource use cost	138	3160 (3869)	157	4845 (8336)
Cost of the UCL Live Well with Parkinson's Intervention	166	284 (66)	–	–
**Total health and social care cost including the Intervention**	**138**	**3455 (3874)**	**157**	**4688 (7470)**
**Adjusted mean difference controlling for baseline values (95% CI)**	**−£1327 (−£2716 to £63)**			
**Adjusted mean difference accounting for correlation with QALYs (95% CI)**	**−£1282 (−£2700 to −£118)**			
Community health services private				
Baseline	166	109 (287)	180	120 (502)
6-months	149	219 (932)	167	151 (651)
12-months	138	170 (879)	159	70 (204)
Total at 12-months	138	377 (1717)	157	227 (707)
Local transport (privately funded)				
Baseline	166	4 (14)	180	6 (23)
6-months	149	2 (9)	167	5 (20)
12-months	138	4 (18)	159	3 (11)
Total at 12-months	138	7 (24)	157	8 (25)
Home adaptations (privately funded)				
Baseline	166	5337 (17,742)	180	5871 (18,490)
6-months	149	3649 (13,324)	167	3843 (12,861)
12-months	138	4133 (16,836)	159	4401 (14,834)
Total at 12-months	138	7169 (21,631)	157	8244 (20,956)
Residential and respite care (private)				
Baseline	166	0 (0)	180	0 (0)
6-months	149	0 (0)	167	2 (23)
12-months	138	0 (0)	159	0 (0)
Total at 12-months	138	0 (0)	157	2 (24)
Personal care and help at home (private)				
Baseline	166	391 (1629)	180	855 (3917)
6-months	149	546 (2171)	167	1686 (18,251)
12-months	138	192 (732)	159	333 (1651)
Total at 12-months	138	715 (2389)	157	588 (1917)
Personal care and help at home (unpaid)				
Baseline	166	3671 (8693)	180	3600 (7783)
6-months	149	3391 (8702)	167	2846 (6756)
12-months	138	3012 (7789)	159	3289 (7900)
Total at 12-months	138	6404 (14,640)	157	6194 (12,278)
Cost of reduced hours (participant with PD)				
Baseline	166	8 (64)	180	9 (106)
6-months	149	2 (24)	167	3 (22)
12-months	138	0 (0)	159	4 (37)
Total at 12-months	138	2 (25)	157	7 (52)
Cost of becoming unemployed due to health issues				
Baseline	166	NA	180	NA
6-months	149	0	167	154 (1992)
12-months	138	NA	159	NA
Total at 12-months	138	NA	157	NA
Cost of days of absence due to illness				
Baseline	166	7822 (19,850)	180	5365 (16,918)
6-months	149	5859 (17,535)	167	5212 (16,510)
12-months	138	5206 (15,077)	159	3659 (13,632)
Total at 12-months	138	11,344 (32,268)	157	8528 (27,359)
Cost of reduced hours (participant's carer)				
Baseline	166	8 (64)	180	9 (106)
6-months	149	2 (24)	167	3 (22)
12-months	138	0 (0)	159	4 (37)
Total at 12-months	138	2 (25)	157	7 (52)
**Total Wider Societal Costs (wider costs + healthcare and social care costs)**	138	14,531 (26,191)	157	16,522 (27,197)
**Adjusted mean difference controlling for baseline values (95% CI)**	**−**£2647 (**−**£8659 to £3366)			
**Adjusted mean difference accounting for correlation with QALYs (95% CI)**	**−**£2682 (**−**£9687 to £2764)			

There was no statistically significant impact on QALYs (0.018; 95% CI (−0.006 to 0.042)). Under a health and social care perspective, there was a 99% probability that the intervention is cost-effective compared to TAU at a decision threshold of £20,000 per QALY and 98% at £30,000 (Supplementary Figures S1 and S2).

The *UCL Live Well with Parkinson's* toolkit remained cost-saving compared to TAU at the maximum and minimum cost per participant, with a mean incremental cost of −£1340 and −£1161 respectively.

The number of adverse events reported was similar in both groups (75 adverse events among 54 participants in the intervention group and 73 events among 49 participants in the TAU group). There were similar numbers of SAEs in both arms (19 among 15 participants in the intervention and 18 among 18 participants in the TAU arm) and none were associated with the intervention.

## Discussion

In the *UCL Live Well with Parkinson's* self-management RCT there was little difference in quality of life (PDQ-39 score) between the intervention group and TAU. However, there were improvements in functional outcomes (MDS-UPDRS part I&II) at 6-months and 12-months in the intervention group compared with the TAU group, reflecting improved experiences of non-motor and motor activities of daily living. These were also reflected on other secondary outcome measures, including the overall non-motor symptom (MDS-NMS) score, particularly its depression and psychosis subdomains, the GHQ-12 score of psychological distress, and participants' own assessment of overall health (EQ-5D-5L VAS score).

Although the clinical primary outcome analysis was negative, with no measurable improvement on the health-related quality of life measure there were positive findings in subgroup analysis and secondary outcomes, and the secondary study aim of health economic evaluation suggested that the intervention was cost saving. There are several possible reasons for the lack of improvement on the health-related quality of life measure despite improvement on functional, non-motor and mental health outcomes. We recruited a relatively high proportion of individuals from underserved populations, due to our targeted recruitment, but the population enrolled was nevertheless largely white, from a higher socio-economic background, with higher education level and from urban areas, in keeping with most research studies. These populations may already have good access to healthcare resources and therefore be less likely to benefit from additional self-management tools. It is therefore of note in our subgroup analysis that we did find a significant improvement in the underserved population, particularly those from rural areas, which are known to have less access to healthcare resources. This suggests that these communities may have more scope for improvement with such a facilitated self-management tool, but this should be replicated in further work. The overall cost-saving of the intervention also supports a benefit and may be most relevant in those underserved communities. Providing such facilitated self-management interventions to those communities may thus be most beneficial. It is also possible that the PDQ-39, which reflects the concept of overall health-related quality of life, is not sensitive to change with such complex multi-component interventions. Despite this limitation, PDQ-39 scores were significantly improved in the subgroup analysis of those from underserved groups (particularly rural areas). As these groups typically have less access to specialist services, this intervention therefore has the potential to be particularly helpful for people in these areas by bridging gaps in support and information needs in between appointments. However, these findings need to be interpreted with caution as the study was not powered to specifically investigate the effects in these subgroups.

The intervention was found to be both cost-saving and likely to be cost-effective under commonly applied thresholds.^29^ This cost reduction was mainly due to a reduction in unplanned admissions, hospital health-care costs as well as home adaptations in the intervention group. Whilst we did not see a reduction in adverse events, these were overall rare and mild in this population. The reduction in hospital admissions suggests that this expected effect may have led to earlier intervention reducing hospital admissions. This reduction in cost alone, together with the high acceptability by the study population,^30^ suggests that implementation of the intervention could also be beneficial also for healthcare service providers, reducing the burden on hospital and care resources. However, it is important to note that this intervention is not a replacement of specialist services but aims to be an additional resource for people with Parkinson's and their carers to help them manage their disease, emerging symptoms, medication queries and symptoms. It meets the aim of enabling patients to manage their own disease rather than being only recipients of care. We aimed to achieve this by provision of information, tailored and personalised goal setting, action planning, problem solving, personalised advice on lifestyle of support, and self-monitoring, supported by trained but not specialised HCPs. In this way it fulfils many of the aims of the World Health Organisation public health approach to PD and the NHS 10-year Plan.^31^^,^^32^ The Plan proposes three radical changes to how health and care is delivered and managed in England: a shift from fragmented hospital-based care to more continuous, integrated community centred care with a focus on disease prevention; increased use of digital tools and technology to ensure rapid access to services; and a focus on disease prevention and self-management. The online *UCL Live Well with Parkinson's* self-management intervention could support these ambitions by enabling people with Parkinson's and carers to identify problems they may be experiencing, take more control of their disease, and support symptom management.

Limitations of this study include predominant recruitment of people with mild to moderate Parkinson's’ disease and those with severe cognitive impairment. Findings may not apply to people with more advanced disease although we did not find any differences by disease severity group. The demographic characteristics of our study population were broadly representative of the general Parkinson's population in England with adequate representation of non-white minority groups in this age group (7%). We also successfully recruited people from underserved populations, and our findings suggest that the intervention may be particularly beneficial in people from underserved groups. However, despite the advantages of this real-world and inclusive approach, it also raises the possibility that the heterogeneity through differences in participant, disease and treatment characteristics introduced by this approach may have diluted or obscured the true effect of the intervention, and remaining recruitment biases can also not be excluded. A limitation in assessment includes the remote administration of outcome measures, including the PDQ-39. This method of administration may have introduced a difference to the standard self-completion format, potentially influencing the results. Also, although the tool can inform communication with healthcare professionals about symptoms, including tracking them in relation to medication over time, it is not designed to provide monitoring of symptoms by the healthcare professionals (or the facilitator) but is a tool enabling self-monitoring to support self-management. Finally, as our aim was to investigate the effectiveness of the intervention compared to usual care in a real-life setting, we did not compare against other interventions. Future studies could investigate the specific effect of this intervention against other active interventions such provision of information only or a more specific intervention such as occupational therapy.

### Conclusion

This is the first large randomised controlled trial on the clinical and cost-effectiveness of a facilitated self-management intervention for people with Parkinson's disease. We did not find an improvement in the primary outcome measure overall (the health-related quality of life measure PDQ-39). However, the intervention improved functional, non-motor and mental health outcomes and was cost-effective.

## Contributors

AS and KW were joint Chief Investigators and were responsible for study conceptualisation, methodology, funding acquisition, supervision, data analysis and interpretation and writing, reviewing and editing the manuscript. AS and KK wrote the first draft of the manuscript and TR, BG, GA, RH, ND, CA, PS, MA, LG, JW, and KW contributed to the manuscript review. All authors contributed to the study oversight. KK was the Programme Manager and responsible for project administration, supervision, literature search and writing and reviewing the manuscript. TR was the Deputy Trial Manager and responsible for project administration, supervision, data collection, analysis and interpretation and reviewing and editing the manuscript. BG, GA, RH, ND, CA, and PS were co-investigators. MC was the public contributor with lived experience and contributed to manuscript review and editing. BG, GA, RH, ND, PS, MA, LG, and JW were involved in data analysis and/or interpretation, data visualisation and reviewing and editing the manuscript. MA did the main statistical analysis, which was reviewed by GA, the senior statistician. RH, LG and JW did the health economic analysis. All authors had full access to all data in the study. KW, AS, MA, GA, and RH independently verified the data. All authors contributed to editing of the manuscript and had full responsibility for the decision to submit for publication.

## Data sharing statement

Aggregated data analysed during the trial are available for scientific review upon reasonable request to the Priment CTU (priment@ucl.ac.uk), after publication of this article. Personal participant data, which are under the responsibility of the sponsor, will be accessible for scientific review only under conditions defined by GDPR. A data transfer agreement will be signed with the sponsor, specifying the scope of data transfer (as required by GDPR) and including an obligation to use the data solely for scientific review purposes and to prohibit disclosure to third parties.

## Declaration of interests

KW has received funding from National Institutes for Health and Care Research (NIHR) programmes and Alzheimer's Society and is a member of the NIHR School of Public Health Research and NIHR Three Schools Prevention Advisory Boards.

AS has received research funding or support from the National Institute of Health, National Institute for Health Research ULCH Biomedical Research Centre, the International Parkinson and Movement Disorder Society, the European Commission and Parkinson's UK and Honoraria for consultancy from Biogen, Abbvie, Bial and Otsuka.

RH has received research funding or support from the National Institute for Health Research (NIHR) Policy Research Unit in Dementia and Neurodegeneration, Research Support Service and Blood Borne Viruses Health protection Research Unit and consultancy fees from QuidelOrtho and University of Nottingham, UK.

All other authors declare no competing interests.

## Appendix A Supplementary data

Supplementary Tables and Figures

Protocol
