# Supplementary material for: Clinical and Cost-Effectiveness of the “UCL Live Well With Parkinson's” Toolkit: A Randomised Controlled Trial
Source: Lancet Reg Health Eur. 2026 Jul 21;68:101762. doi: 10.1016/j.lanepe.2026.101762 (PMC13392959; doi:10.1016/j.lanepe.2026.101762)
Supplement: Supplementary Tables and Figures [file mmc1.docx]

# Supplementary material

Supplementary Figure 1: Cost-effectiveness acceptability curves (CEAC)


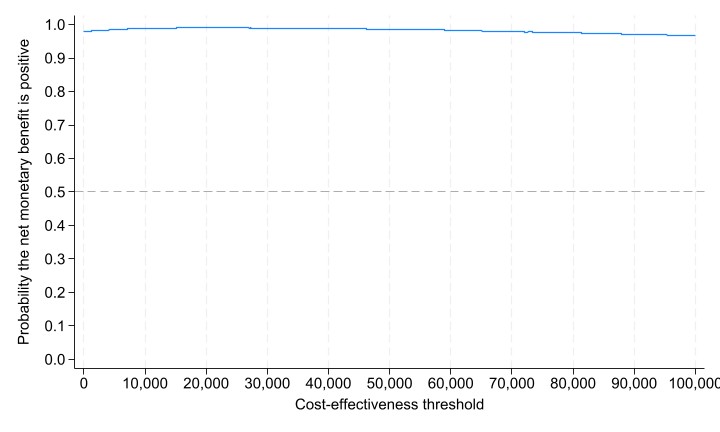


Supplementary Figure 2: Cost-effectiveness planes (CEP)


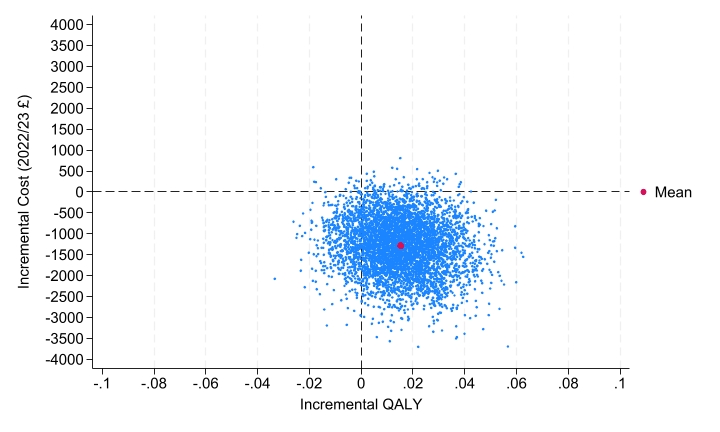


Supplementary Table 1: Number and percentage of participants from underserved populations

|  | TAU (n=180) | Intervention (n=166) | Total (n=346) |
| --- | --- | --- | --- |
| Non- white ethnic group | 16 (9%) | 9 (5%) | 25 (7%) |
| Left education before age 16 | 24 (13%) | 17 (10%) | 41 (12%) |
| Deprivation Index 1-3 | 47 (26%) | 32 (19%) | 79 (23%) |
| Living in a rural area | 27 (15%) | 34 (20%) | 61 (18%) |
| At least one underserved group (including rurality) | 93 (52%) | 75 (45%) | 168 (49%) |
| At least one underserved group (excluding rurality) | 70 (39%) | 50 (30%) | 120 (35%) |

Supplementary Table 2: Results of subgroup analysis of PDQ-39 for underserved subgroups

|  | Treatment as usual | | Intervention | | Adjusted mean difference (95% CI) | p-value for interaction test |
| --- | --- | --- | --- | --- | --- | --- |
|  | n | Mean (SD) | n | Mean (SD) |  |  |
| Baseline |  |  |  |  |  |  |
| At least one underserved group (including rurality) | 93 | 25.1 (14.7) | 75 | 24.6 (15.5) |  |  |
| Not in any underserved group (including rurality) | 87 | 20.7 (12.9) | 91 | 20.4 (12.6) |  |  |
| 6-month |  |  |  |  |  | 0.45 |
| At least one underserved group (including rurality) | 83 | 24.9 (15.3) | 70 | 23.0 (16.2) | -2.0 (-4.9 to 0.8) |  |
| Not in any underserved group (including rurality) | 85 | 19.9 (13.8) | 79 | 19.3 (12.0) | -0.5 (-3.3 to 2.2) |  |
| 12-month |  |  |  |  |  | 0.01 |
| At least one underserved group (including rurality) | 80 | 26.6 (16.0) | 65 | 21.9 (14.9) | -4.0 (-6.8 to -1.1) |  |
| Not in any underserved group (including rurality) | 84 | 20.5 (13.1) | 76 | 21.9 (15.0) | 1.8 (-0.9 to 4.5) |  |
| Baseline |  |  |  |  |  |  |
| At least one underserved group (excluding rurality) | 70 | 26.0 (15.7) | 50 | 24.1 (16.1) |  |  |
| Not in any underserved group (excluding rurality) | 110 | 21.1 (12.5) | 116 | 21.5 (13.2) |  |  |
| 6-month |  |  |  |  |  | 0.73 |
| At least one underserved group (excluding rurality) | 61 | 25.0 (16.5) | 46 | 22.7 (16.7) | -1.7 (-5.2 to 1.7) |  |
| Not in any underserved group (excluding rurality) | 107 | 20.9 (13.5) | 103 | 20.2 (12.9) | -1.0 (-3.4 to 1.4) |  |
| 12-month |  |  |  |  |  | 0.07 |
| At least one underserved group (excluding rurality) | 60 | 26.6 (16.3) | 43 | 21.7 (16.1) | -3.6 (-7.1 to -0.2) |  |
| Not in any underserved group (excluding rurality) | 104 | 21.7 (13.7) | 98 | 21.9 (14.4) | 0.3 (-2.1 to 2.8) |  |
| Baseline |  |  |  |  |  |  |
| Living in a rural area | 27 | 22.8 (11.5) | 34 | 23.1 (13.8) |  |  |
| Not living in rural area | 153 | 23.0 (14.4) | 132 | 22.1 (14.2) |  |  |
| 6-month |  |  |  |  |  | 0.45 |
| Living in a rural area | 24 | 23.6 (11.7) | 33 | 20.8 (14.4) | -3.0 (-7.7 to 1.7) |  |
| Not living in rural area | 144 | 22.2 (15.2) | 116 | 21.0 (14.2) | -1.0 (-3.2 to 1.2) |  |
| 12-month |  |  |  |  |  | 0.04 |
| Living in a rural area | 22 | 26.4 (14.6) | 29 | 19.9 (12.0) | -5.7 (-10.5 to -0.9) |  |
| Not living in rural area | 142 | 23.0 (14.9) | 112 | 22.4 (15.6) | -0.2 (-2.3 to 2.0) |  |
| Baseline |  |  |  |  |  |  |
| Deprivation Index 1-3 | 47 | 27.3 (14.7) | 32 | 25.7 (18.1) |  |  |
| Deprivation index 4-6 | 133 | 21.4 (13.5) | 133 | 21.2 (12.5) |  |  |
| 6-month |  |  |  |  |  | 0.98 |
| Deprivation Index 1-3 | 43 | 24.9 (13.7) | 28 | 23.6 (18.6) | -1.2 (-5.5 to 3.1) |  |
| Deprivation index 4-6 | 125 | 21.6 (15.0) | 120 | 20.2 (12.8) | -1.3 (-3.5 to 1.0) |  |
| 12-month |  |  |  |  |  | 0.13 |
| Deprivation Index 1-3 | 41 | 26.4 (14.4) | 28 | 22.1 (17.5) | -4.0 (-8.2 to 0.2) |  |
| Deprivation index 4-6 | 123 | 22.5 (14.9) | 112 | 21.5 (14.1) | -0.3 (-2.6 to 1.9) |  |
| Baseline |  |  |  |  |  |  |
| White | 16 | 29.4 (20.3) | 9 | 27.0 (15.2) |  |  |
| Non-white | 164 | 22.4 (13.2) | 157 | 22.0 (14.0) |  |  |
| 6-month |  |  |  |  |  | 0.38 |
| White | 13 | 32.6 (21.9) | 9 | 28.6 (20.8) | -4.4 (-11.9 to 3.1) |  |
| Non-white | 155 | 21.6 (13.7) | 140 | 20.5 (13.6) | -0.9 (-2.9 to 1.1) |  |
| 12-month |  |  |  |  |  | 0.46 |
| White | 13 | 32.0 (21.4) | 8 | 28.1 (17.4) | -3.8 (-11.5 to 4.0) |  |
| Non-white | 151 | 22.7 (14.0) | 133 | 21.5 (14.7) | -0.8 (-2.8 to 1.3) |  |
| Baseline |  |  |  |  |  |  |
| Left Education <16 | 24 | 24.9 (16.9) | 17 | 24.8 (17.2) |  |  |
| Left Education ≥16 | 156 | 22.7 (13.5) | 149 | 22.0 (13.8) |  |  |
| 6-month |  |  |  |  |  |  |
| Left Education <16 |  |  |  |  |  | 0.30 |
| Left Education ≥16 | 20 | 21.8 (14.1) | 17 | 23.3 (16.6) | 1.6 (-4.2 to 7.4) |  |
| 12-month | 148 | 22.5 (14.9) | 132 | 20.7 (13.9) | -1.7 (-3.8 to 0.4) |  |
| Left Education <16 | 19 | 25.2 (18.1) | 15 | 22.0 (16.2) | -2.4 (-8.3 to 3.6) |  |
| Left Education ≥16 | 145 | 23.3 (14.4) | 126 | 21.8 (14.8) | -0.9 (-3.0 to 1.2) |  |

Supplementary Table 3: Results of subgroup analysis of PDQ-39 for disease severity (MDS-UPDRS part I&II)

|  | Treatment as usual | | Intervention | | Adjusted mean difference (95% CI) | p-value for interaction test |
| --- | --- | --- | --- | --- | --- | --- |
|  | n | Mean (SD) | n | Mean (SD) |  |  |
| Baseline |  |  |  |  |  |  |
| Mild | 90 | 14.0 (8.5) | 91 | 14.0 (8.3) |  |  |
| Moderate | 85 | 31.0 (12.2) | 70 | 31.0 (11.7) | - | - |
| Severe | 5 | 48.8 (5.0) | 4 | 59.4 (7.7) |  |  |
| 6-month |  |  |  |  |  | 0.18 |
| Mild | 87 | 13.9 (8.7) | 80 | 13.6 (9.5) | -0.02 (-2.71 to 2.67) |  |
| Moderate | 77 | 31.1 (14.6) | 64 | 28.0 (12.6) | -2.92 (-5.87 to 0.03) |  |
| Severe | 4 | 41.0 (5.1) | 4 | 54.8 (9.2) | 6.26 (-6.05 to 18.57) |  |
| 12-month |  |  |  |  |  | 0.98 |
| Mild | 83 | 14.6 (9.3) | 77 | 13.5 (9.5) | -0.96 (-3.70 to 1.78) |  |
| Moderate | 77 | 32.0 (13.9) | 60 | 31.1 (13.7) | -0.90 (-3.90 to 2.09) |  |
| Severe | 4 | 44.5 (7.7) | 3 | 51.8 (6.4) | 0.55 (-12.59 to 13.69) |  |

Supplementary Table 4: MDS-NMS scale sub-domain scores at baseline, 6- and 12-months

|  | Treatment as usual | | Intervention | | Adjusted mean difference (95% CI) |
| --- | --- | --- | --- | --- | --- |
|  | n | Mean (SD) | n | Mean (SD) |  |
| A – Depression |  |  |  |  |  |
| Baseline | 178 | 7.85 (11.61) | 163 | 8.56 (12.72) |  |
| 6-month | 162 | 9.04 (12.77) | 145 | 6.83 (9.36) | **-3.38 (-5.26 to -1.49)** |
| 12-month | 157 | 8.79 (12.18) | 134 | 6.69 (10.66) | **-2.44 (-4.55 to -0.33)** |
| B – Anxiety |  |  |  |  |  |
| Baseline | 178 | 7.74 (9.55) | 163 | 7.74 (9.78) |  |
| 6-month | 164 | 8.18 (9.06) | 145 | 7.40 (8.09) | -1.33 (-2.76 to 0.10) |
| 12-month | 157 | 7.32 (7.97) | 133 | 7.08 (9.15) | -0.58 (-2.21 to 1.06) |
| C – Apathy |  |  |  |  |  |
| Baseline | 178 | 5.15 (7.31) | 163 | 5.31 (7.56) |  |
| 6-month | 164 | 4.76 (6.72) | 143 | 4.27 (6.16) | -0.62 (-1.86 to 0.62) |
| 12-month | 157 | 5.10 (7.27) | 135 | 4.50 (7.02) | -0.78 (-2.25 to 0.70) |
| D – Psychosis |  |  |  |  |  |
| Baseline | 178 | 1.73 (4.37) | 163 | 1.63 (4.69) |  |
| 6-month | 163 | 1.47 (4.01) | 145 | 1.08 (3.86) | -0.31 (-0.99 to 0.36) |
| 12-month | 157 | 1.28 (3.63) | 134 | 0.65 (2.81) | **-0.72 (-1.39 to -0.06)** |
| E – Impulse control and related disorders |  |  |  |  |  |
| Baseline | 178 | 0.72 (2.31) | 163 | 1.09 (4.20) |  |
| 6-month | 164 | 0.75 (2.06) | 145 | 0.87 (2.91) | -0.11 (-0.51 to 0.29) |
| 12-month | 157 | 0.39 (1.17) | 135 | 0.63 (2.32) | 0.08 (-0.23 to 0.39) |
| F – cognition |  |  |  |  |  |
| Baseline | 178 | 11.58 (13.89) | 162 | 11.57 (15.37) |  |
| 6-month | 150 | 10.53 (12.27) | 134 | 10.72 (12.99) | -0.44 (-2.44 to 1.56) |
| 12-month | 135 | 12.55 (13.89) | 112 | 11.89 (14.57) | -0.38 (-3.17 to 2.41) |
| G – Orthostatic Hypotension |  |  |  |  |  |
| Baseline | 178 | 3.19 (5.02) | 163 | 3.11 (5.81) |  |
| 6-month | 164 | 2.86 (4.83) | 145 | 2.63 (4.83) | -0.26 (-1.22 to 0.70) |
| 12-month | 157 | 3.14 (4.84) | 135 | 2.61 (4.59) | -0.44 (-1.43 to 0.55) |
| H – Urinary |  |  |  |  |  |
| Baseline | 178 | 10.63 (11.85) | 163 | 10.55 (12.61) |  |
| 6-month | 163 | 10.90 (11.34) | 145 | 9.75 (11.09) | -1.58 (-3.35 to 0.20) |
| 12-month | 156 | 10.70 (11.66) | 133 | 9.29 (10.88) | -1.38 (-3.46 to 0.70) |
| I – Sexual |  |  |  |  |  |
| Baseline | 173 | 4.53 (7.83) | 157 | 5.52 (8.55) |  |
| 6-month | 155 | 3.95 (7.44) | 139 | 4.86 (7.84) | -0.00 (-1.39 to 1.38) |
| 12-month | 145 | 2.90 (5.84) | 128 | 4.11 (7.11) | 0.58 (-0.69 to 1.84) |
| J – Gastrointestinal |  |  |  |  |  |
| Baseline | 178 | 7.93 (8.69) | 163 | 6.21 (6.84) |  |
| 6-month | 164 | 7.80 (8.51) | 145 | 6.47 (7.71) | -0.30 (-1.72 to 1.11) |
| 12-month | 157 | 8.32 (8.88) | 135 | 5.97 (6.82) | -1.28 (-2.76 to 0.20) |
| K – Sleep and wakefulness |  |  |  |  |  |
| Baseline | 178 | 12.30 (11.19) | 163 | 13.19 (12.61) |  |
| 6-month | 163 | 11.80 (10.12) | 145 | 11.68 (10.98) | -0.57 (-2.56 to 1.42) |
| 12-month | 155 | 12.63 (10.33) | 134 | 11.45 (9.91) | -1.57 (-3.54 to 0.40) |
| L – Pain |  |  |  |  |  |
| Baseline | 177 | 11.80 (10.85) | 163 | 11.48 (11.14) |  |
| 6-month | 163 | 11.10 (10.73) | 145 | 10.37 (11.30) | -1.08 (-3.00 to 0.83) |
| 12-month | 156 | 10.24 (9.48) | 135 | 9.34 (8.81) | -0.93 (-2.75 to 0.90) |
| M – Other |  |  |  |  |  |
| Baseline | 177 | 9.89 (10.01) | 163 | 10.35 (11.32) |  |
| 6-month | 162 | 12.12 (10.90) | 143 | 11.71 (11.94) | -1.13 (-3.21 to 0.95) |
| 12-month | 156 | 12.67 (11.93) | 134 | 11.62 (10.80) | -1.55 (-3.88 to 0.78) |

Supplementary Table 5: Carer outcomes at baseline 6- and 12-months

| Outcome | Treatment as usual | | Intervention | | Adjusted mean difference (95% CI) |
| --- | --- | --- | --- | --- | --- |
|  | n | Mean (SD) | n | Mean (SD) |  |
| Zarit score |  |  |  |  |  |
| Baseline | 81 | 18.85 (12.98) | 65 | 18.49 (12.67) |  |
| 6-month | 68 | 20.01 (14.10) | 52 | 18.79 (15.09) | -0.14 (-3.06 to 2.78) |
| 12-month | 64 | 22.97 (14.02) | 55 | 23.55 (15.83) | 1.49 (-2.23 to 5.22) |
| Carer QOL score |  |  |  |  |  |
| Baseline | 81 | 18.61 (17.80) | 66 | 18.79 (16.97) |  |
| 6-month | 70 | 20.48 (17.75) | 53 | 21.26 (19.82) | 0.70 (-2.55 to 3.95) |
| 12-month | 62 | 23.48 (18.88) | 54 | 25.07 (20.85) | 1.52 (-2.71 to 5.75) |

Supplementary Table 6: Cost of intervention

|  | N | Mean (SD) |
| --- | --- | --- |
| **Number of sessions** | **165** | **5 (2)** |
| **Cost of PD care** | **166** | **284.44 (66)** |
| Cost of consumables per participant | 166 | 61.74 |
| Cost of training, supervision, and line management per participant | 166 | 40.91 |
| Cost of delivery of the intervention | 166 | 188.50 (61.57) |

Supplementary Table 7: Self-reported resource use across all timepoints for participants with PD

|  | Intervention | | Control | |
| --- | --- | --- | --- | --- |
|  | N (%) | Mean (SD) | N (%) | Mean (SD) |
| Emergency services contacts | | | | |
| Baseline | 16 (10%) | 2 (1) | 21 (12%) | 2 (1) |
| 6-months | 13 (9%) | 1 (0) | 19 (11%) | 2 (1) |
| 12-months | 9 (7%) | 1 (0) | 19 (12%) | 2 (1) |
| 12-months total | 19 (14%) | 1 (0) | 31 (20%) | 2 (2) |
| Hospital admissions contacts | | | | |
| Baseline | 6 (4%) | 4 (3) | 10 (6%) | 4 (4) |
| 6-months | 7 (5%) | 4 (3) | 7 (4%) | 4 (2) |
| 12-months | 4 (3%) | 2 (1) | 8 (5%) | 15 (19) |
| 12-months total | 10 (7%) | 3 (3) | 12 (8%) | 12 (16) |
| Specialist visits contacts | | | | |
| Baseline | 129 (78%) | 2 (2) | 133 (74%) | 2 (2) |
| 6-months | 101 (68%) | 2 (2) | 109 (65%) | 2 (1) |
| 12-months | 92 (67%) | 2 (2) | 112 (70%) | 2 (1) |
| 12-months total | 124 (90%) | 3 (3) | 136 (87%) | 3 (2) |
| Primary care and community contacts | | | | |
| Baseline | 115 (69%) | 3 (4) | 126 (70%) | 4 (5) |
| 6-months | 111 (74%) | 3 (3) | 129 (77%) | 3 (3) |
| 12-months | 91 (66%) | 4 (4) | 103 (65%) | 3 (3) |
| 12-months total | 119 (86%) | 5 (6) | 139 (89%) | 5 (5) |
| Number of home adaptations | | | | |
| Baseline | 8 (5%) | 16 (7) | 10 (6%) | 21 (11) |
| 6-months | 3 (2%) | 17 (8) | 10 (6%) | 14 (9) |
| 12-months | 4 (3%) | 20 (11) | 11 (7%) | 14 (7) |
| 12-months total | 6 (4%) | 18 (10) | 17 (11%) | 16 (13) |
| Number of personal help contacts | | | | |
| Baseline | 2 (1%) | 27 (38) | 2 (1%) | 5 (0) |
| 6-months | 1 (1%) | 54 (0) | 2 (1%) | 80 (80) |
| 12-months | 3 (2%) | 143 (128) | 0 (0%) | 0 (0) |
| 12-months total | 4 (3%) | 120 (113) | 2 (1%) | 80 (80) |
| Number of contacts for Community Transport | | | | |
| Baseline | 1 (1%) | 3 (0) | 1 (1%) | 1 (0) |
| 6-months | 1 (1%) | 2 (0) | 1 (1%) | 4 (0) |
| 12-months | 0 (0%) | 0 (0) | 1 (1%) | 2 (0) |
| 12-months total | 1 (1%) | 2 (0) | 2 (1%) | 3 (1) |
| Number of people using medications | | | | |
| Baseline | 163 (98%) | 1 (0) | 177 (98%) | 1 (0) |
| 6-months | 148 (99%) | 1 (0) | 165 (99%) | 1 (0) |
| 12-months | 138 (100%) | 1 (0) | 156 (98%) | 1 (0) |
| 12-months total | 138 (100%) | 2 (0) | 156 (99%) | 2 (0) |
| Primary and community care private contacts | | | | |
| Baseline | 75 (45%) | 11 (17) | 70 (39%) | 9 (10) |
| 6-months | 65 (39%) | 8 (12) | 67 (37%) | 10 (11) |
| 12-months | 44 (27%) | 7 (9) | 48 (27%) | 5 (9) |
| 12-months total | 79 (48%) | 10 (13) | 91 (51%) | 10 (13) |
| Personal help privately funded in hours | | | | |
| Baseline | 25 (15%) | 54 (76) | 39 (22%) | 83 (167) |
| 6-months | 29 (17%) | 76 (119) | 32 (18%) | 242 (1152) |
| 12-months | 17 (10%) | 41 (41) | 33 (18%) | 42 (93) |
| 12-months total | 32 (19%) | 90 (114) | 47 (26%) | 195 (951) |
| Personal help unpaid in hours | | | | |
| Baseline | 52 (31%) | 208 (212) | 64 (36%) | 179 (204) |
| 6-months | 40 (24%) | 286 (323) | 46 (26%) | 236 (244) |
| 12-months | 34 (20%) | 283 (322) | 46 (26%) | 279 (296) |
| 12-months total | 50 (30%) | 421 (491) | 65 (36%) | 364 (390) |
| Privately funded transport to attend hospital appointments | | | | |
| Baseline | 29 (17%) | 3 (3) | 32 (18%) | 3 (4) |
| 6-months | 22 (15%) | 3 (2) | 31 (19%) | 3 (2) |
| 12-months | 24 (17%) | 3 (3) | 28 (18%) | 4 (4) |
| 12-months total | 38 (28%) | 4 (3) | 46 (29%) | 4 (4) |
| Number of home adaptations privately paid | | | | |
| Baseline | 19 (11%) | 19 (10) | 23 (13%) | 21 (9) |
| 6-months | 15 (10%) | 18 (6) | 24 (14%) | 15 (9) |
| 12-months | 12 (9%) | 20 (9) | 18 (11%) | 17 (9) |
| 12-months total | 23 (17%) | 20 (10) | 37 (24%) | 17 (11) |

Mean and SD are only for people who used the service (non-zeros)

Supplementary Table 8: PDQ-39 sub-domain analysis at baseline, 6 and 12-months

|  | Treatment as usual | | Intervention | | Adjusted mean difference (95% CI) |
| --- | --- | --- | --- | --- | --- |
|  | n | Mean (SD) | n | Mean (SD) |  |
| Mobility |  |  |  |  |  |
| Baseline | 180 | 29.11 (26.94) | 166 | 27.65 (26.41) |  |
| 6-month | 169 | 29.35 (27.01) | 149 | 26.45 (27.82) | -2.16 (-5.70 to 1.38) |
| 12-month | 164 | 32.17 (29.11) | 141 | 27.59 (27.17) | -2.38 (-6.00 to 1.23) |
| Activity and daily living |  |  |  |  |  |
| Baseline | 180 | 27.29 (21.88) | 166 | 26.73 (23.08) |  |
| 6-month | 169 | 27.22 (22.18) | 149 | 26.59 (23.03) | -0.49 (-3.91 to 2.93) |
| 12-month | 164 | 30.99 (23.68) | 141 | 27.36 (21.82) | -3.23 (-6.93 to 0.47) |
| Emotional |  |  |  |  |  |
| Baseline | 180 | 23.63 (18.71) | 166 | 21.29 (17.78) |  |
| 6-month | 168 | 23.04 (20.69) | 149 | 20.64 (18.45) | -1.32 (-4.62 to 1.97) |
| 12-month | 164 | 24.47 (19.79) | 141 | 21.13 (20.86) | -2.08 (-5.57 to 1.42) |
| Stigma |  |  |  |  |  |
| Baseline | 180 | 15.83 (19.58) | 166 | 15.55 (19.52) |  |
| 6-month | 168 | 15.44 (21.36) | 149 | 13.34 (19.60) | -2.03 (-5.38 to 1.33) |
| 12-month | 164 | 12.61 (19.40) | 141 | 13.98 (20.49) | 1.24 (-2.62 to 5.10) |
| Social support |  |  |  |  |  |
| Baseline | 180 | 10.23 (19.04) | 166 | 8.28 (15.41) |  |
| 6-month | 168 | 8.95 (15.55) | 149 | 6.18 (13.49) | -2.21 (-4.89 to 0.47) |
| 12-month | 164 | 8.79 (17.03) | 141 | 8.27 (16.53) | 0.18 (-2.68 to 3.05) |
| Cognition |  |  |  |  |  |
| Baseline | 180 | 25.32 (18.33) | 166 | 24.22 (19.60) |  |
| 6-month | 168 | 24.55 (17.86) | 149 | 23.49 (18.82) | -0.64 (-3.59 to 2.30) |
| 12-month | 164 | 26.68 (20.20) | 141 | 25.13 (19.73) | -0.28 (-3.66 to 3.10) |
| Communication |  |  |  |  |  |
| Baseline | 180 | 17.08 (18.90) | 166 | 18.72 (21.66) |  |
| 6-month | 169 | 16.27 (17.46) | 149 | 17.84 (19.57) | 0.30 (-2.70 to 3.30) |
| 12-month | 164 | 17.33 (18.83) | 141 | 18.74 (19.97) | 0.70 (-2.56 to 3.95) |
| Bodily discomfort |  |  |  |  |  |
| Baseline | 180 | 35.37 (23.45) | 166 | 35.89 (25.04) |  |
| 6-month | 169 | 34.66 (25.27) | 149 | 33.45 (23.51) | -1.66 (-5.80 to 2.48) |
| 12-month | 164 | 34.81 (24.94) | 141 | 32.68 (22.21) | -2.28 (-6.61 to 2.05) |

Supplementary Table 9: Impact of toolkit mode on primary outcome at 6 and 12-months

|  | Adjusted mean difference (95% CI) |
| --- | --- |
| 6-month |  |
| Paper vs TAU | -0.25 (-3.60 to 3.10) |
| Online vs TAU | -1.49 (-3.63 to 0.65) |
| 12-month |  |
| Paper vs TAU | -1.04 (-4.47 to 2.38) |
| Online vs TAU | -1.09 (-3.27 to 1.08) |

Supplementary Table 10. Characteristics of participants who used the paper toolkit

|  | All (n=39) |
| --- | --- |
| Gender |  |
| Male | 22 (56.4%) |
| Female | 17 (43.6%) |
| Age – mean (SD) | 73.3 (8.8) |
| Ethnicity |  |
| White | 36 (92.3%) |
| Asian/Asian British | 1 (2.6%) |
| Black/African/Black Caribbean/Black British | 1 (2.6%) |
| Other ethnic group | 1 (2.6%) |
| Marital status |  |
| Single/Unmarried | 2 (5.1%) |
| Cohabiting | 1 (2.6%) |
| Married/Civil Partnership | 31 (79.5%) |
| Widowed | 4 (10.3%) |
| Divorced | 1 (2.6%) |
| Residence |  |
| Major conurbation | 6 (15.4%) |
| City (City with abundant services) | 13 (33.3%) |
| Town | 14 (35.9%) |
| Village | 6 (15.4%) |
| IMD decile |  |
| 1-3 (most deprived) | 7 (17.9%) |
| 4-6 | 17 (43.6%) |
| 7-10 (least deprived) | 15 (38.5%) |
| Living arrangements |  |
| Live with spouse/life-partner | 26 (66.7%) |
| Live with family | 5 (12.8%) |
| Live alone | 8 (20.5%) |
| Employment Status |  |
| Full-time employed | 1 (2.6%) |
| Part-time employed | 2 (5.1%) |
| Unable to work due to illness | 2 (5.1%) |
| Self-employed | 1 (2.6%) |
| Retired | 33 (84.6%) |
| Age left education |  |
| <16 | 4 (10.3%) |
| 16-17 | 15 (38.5%) |
| 18-25 | 17 (43.6%) |
| 26≤ | 3 (7.7%) |
| Hoeh and Yahr stage |  |
| 1 | 4 (10.3%) |
| 2 | 2 (5.1%) |
| 3 | 11 (28.2%) |
| 4 | 4 (10.3%) |
| Unable to rate | 18 (46.2%) |

Supplementary Table 11: Results of CACE analysis of primary outcome

|  | Treatment as usual | | Intervention | | Adjusted mean difference (95% CI) | p-value |
| --- | --- | --- | --- | --- | --- | --- |
|  | N | Mean (SD) | n | Mean (SD) |  |  |
| PDQ-39 |  |  |  |  |  |  |
| 12-months | 164 | 23.48 (14.82) | 141 | 21.86 (14.92) | -1.19 (-2.84 to 0.45) | 0.15 |

Supplementary Table 12: QALYs derived from EQ-5D-5L and mapped to EQ-5D-3L, and YFCs derived from the ICECAP-O

| **EQ-5D-5L PD’s participant’s complete case – Mapped to the EQ-5D-3L (Hernandez et al)** | | | | |
| --- | --- | --- | --- | --- |
| Baseline utility | 166 | 0.626 (0.197) | 180 | 0.630 (0.232) |
| 6-months utility | 150 | 0.628 (0.200) | 170 | 0.622 (0.237) |
| 12-months utility | 144 | 0.615 (0.222) | 165 | 0.590 (0.256) |
| 12-months QALYs | 140 | 0.623 (0.190) | 162 | 0.617 (0.215) |
| Adjusted mean difference controlling for baseline | 0·018  (-0.006 to 0.042) |  | p-value | 0.137 |
| Adjusted mean difference accounting for correlations with costs | 0.015 (-0.012 to 0.041) |  | p-value | 0.266 |
| **ICECAP-O PD’s participants complete case** | | | | |
| Baseline utility | 166 | 0.791 (0.136) | 180 | 0.796 (0.132) |
| 6-months utility | 148 | 0.797 (0.142) | 169 | 0.796 (0.124) |
| 12-months utility | 142 | 0.795 (0.133) | 158 | 0.773 (0.153) |
| 12-months QALYs | 138 | 0.796 (0.130) | 155 | 0.795 (0.114) |
| Adjusted mean difference controlling for baseline | 0.010 (-0.006 to 0.025) |  | p-value | 0.215 |
| Adjusted mean difference accounting for correlations with costs | 0.055 (-0.012 to 0.019) |  | p-value | 0.598 |

Supplementary Table 13: Cost-effectiveness output using Seemingly Unrelated Regression (SUREG) using ICECAP-O

|  | Mean | 95% CI |  | Mean | 95% CI |
| --- | --- | --- | --- | --- | --- |
| Difference in health care and social care costs | -£1,104 | -£2,337 to £65 | Difference in wider, health care, and social care costs | -£2,748 | -£9,505 to £2,901 |
| Difference in Years of Full Capability (YFC) | 0.055 | (-0.012 to 0.019) | Difference in Years of Full Capability (YFC) | 0.053 | (-0.012 to 0.018) |

Supplementary Table 14: Unit costs

| **Variable name** | **Unit Cost, £ (2022/2023)** | **Unit** | **Source** | **Notes/Assumptions** |
| --- | --- | --- | --- | --- |
| GP Consultation at surgery | 49 | per surgery consultation | PSSRU 2023.  Table 9.4.2 pg 64 | GP consultation multiplied by surgery: clinic ratio.  This cost is based on GP consultation of an average 10 minutes per consultation |
| GP telephone consultation | 24.5 | per e-consultation |  | GP consultation multiplied by surgery: phone ratio. |
| GP at home consultation | 73.5 | per home consultation |  | GP consultation multiplied by surgery: home ratio |
| Nurse at the GP practice (Practice nurse) | 53 | per consultation | PSSRU 2023. Table 9.2.1, pg 61 Table 9.3.1 pg 68 | Cost per working hour including qualifications. |
| District Nurse | 11 | per consultation | PSSRU 2023.  Table 9.3.1 pg 68 | Hourly wage of a band 6 nurse multiplied by surgery appointment (10 minutes) time from PSSRU 2023. |
| Health Care Assistant | 36 | per consultation | PSSRU 2023.  Table 8.2.1 pg 56 | Cost per working hour including qualifications |
| Occupational therapist | 68 | per consultation | NHS Reference Costs 2022/2023 |  |
| Physiotherapist one-to-one | 41 | per consultation | NHS Reference Costs 2022/2023 |  |
| Group exercise class e.g. for falls, balance or muscle strength | 116 | per consultation | NHS Reference Costs 2022/2023 |  |
| Speech and language therapist | 100 | per consultation | NHS Reference Costs 2022/2023 |  |
| Optician/Optometrist | 63.9 | per e-consultation | NHS Reference Costs 2022/2023 |  |
| Counsellor or therapist one-to-one | 99 | per consultation | NHS Reference Costs 2022/2023 |  |
| Social worker face-to-face | 53 | per consultation | PSSRU 2023 |  |
| Social worker phone call | 26·5 | per consultation | PSSRU 2023 |  |
| Dentist | 147 | per consultation | PSSRU 2023 | Per hour of patient contact time |
| Podiatrist or chiropodist | 36 | per consultation | NHS Reference Costs 2022/2023 |  |
| Chiropractor or osteopath | 123 | Per item | NHS Reference Costs 2022/2023 |  |
| Bath board | 26 | Per item | PSSRU 2013. Table 7.3.1, pg 109. Uprated to 2023. |  |
| Shower chair | 66.56 | Per item | PSSRU 2013. Table 7.3.1, pg 109. Uprated to 2023. |  |
| Over toilet frame / raised seat | 33 | Per item | PSSRU 2013. Table 7.3.1, pg 109. Uprated to 2023. |  |
| Bathroom stool | 98.03 | Per item | [Living Made Easy - Home](https://livingmadeeasy.org.uk/).  Accessed: December, 2024. |  |
| Bed rail/lever | 38.73 | Per item | [Living Made Easy - Home](https://livingmadeeasy.org.uk/).  Accessed: December, 2024. |  |
| Hospital/electric bed | 995 | per item | [Living Made Easy - Home](https://livingmadeeasy.org.uk/).  Accessed: December, 2024. |  |
| Raiser recliner chair | 220 | per item | [Living Made Easy - Home](https://livingmadeeasy.org.uk/).  Accessed: December, 2024. |  |
| Memory aids | 118 | per item | [Living Made Easy - Home](https://livingmadeeasy.org.uk/).  Accessed: December, 2024. |  |
| Care alarm | 29 | per item | [Living Made Easy - Home](https://livingmadeeasy.org.uk/).  Accessed: December, 2024. |  |
| Walking stick | 15.37 | per item | [Living Made Easy - Home](https://livingmadeeasy.org.uk/).  Accessed: December, 2024. |  |
| Wheelchair | 290 | per item | PSSRU 2023, pg 37 |  |
| Walking frame | 22 | per item | [Living Made Easy - Home](https://livingmadeeasy.org.uk/).  Accessed: December, 2024. |  |
| Walker with wheels | 48 | per item | [Living Made Easy - Home](https://livingmadeeasy.org.uk/).  Accessed: December, 2024. |  |
| Mobility scooter | 1080 | per item | [Living Made Easy - Home](https://livingmadeeasy.org.uk/).  Accessed: December, 2024. |  |
| Banana board/ slide board | 79 | per item | [Living Made Easy - Home](https://livingmadeeasy.org.uk/).  Accessed: December, 2024. |  |
| Emergency call (999) | 12 | per call | Turner J, et al (2021).  Impact of NHS 111 Online  on the NHS 111 telephone  service and urgent  care system: a  mixed methods  study. Health Serv Deliv Res. | No information on cost of NHS call cost available on PSSRU, so used information on the Turner J et al (2021) study. Uprated to 2023. |
| NHS Direct of CALL 111 | 12 | Per call | Turner J, et al (2021).  Impact of NHS 111 Online  on the NHS 111 telephone  service and urgent  care system: a  mixed methods  study. Health Serv Deliv Res. | No information on cost of NHS call cost available on PSSRU, so used information on the Turner J et al (2021) study. Uprated to 2023. |
| Paramedic only | 327 | per service | NHS reference cost- EC services. 2022/2023 |  |
| Paramedic and ambulance to hospital | 459 | per service | NHS reference cost- EC services. 2022/2023 |  |
| A&E attendance without ambulance | 129 | per service | NHS reference cost- EC services. 2022/2023 |  |
| Unplanned (emergency) inpatient hospital stay (short vs long) | 790/5177 | Per visit | NHS reference cost- EC services. 2022/2023 |  |
| Planned (elective) inpatient hospital stay | 6257 | Per visit | NHS reference cost- EC services. 2022/2023 |  |
| Specialised rehabilitation service overnight stay | 130 | Per stay | NHS reference cost- EC services. 2022/2023 |  |
| Local or district rehabilitation service overnight stay | 342 | Per stay | NHS reference cost- EC services. 2022/2023 |  |
| Local authority residential care/care home for respite | 247 | Per call | NHS reference cost- EC services. 2022/2023 |  |
| Private or independent sector residential care/care home | 148 | per service | PSSRU table 6.1.1 pg40 2023 |  |
| Nursing home for respite | 247 | per service | Average national cost  2022/2023 (NHS reference cost) |  |
| Extra care (sheltered) housing | 247 | per service | NHS reference cost  MH services 2022/2023 |  |
| Neurology outpatient appointment | 395 | per service | NHS reference cost- EC services. 2022/2023 |  |
| Geriatrics outpatient appointment | 278 | per service | NHS reference cost- EC services. 2022/2023 |  |
| Urology or continence appointment | 190 | per service | NHS reference cost- EC services. 2022/2023 |  |
| Psychiatric outpatient appointment | 175 | per service | NHS reference cost- EC services. 2022/2023 |  |
| Hospital memory clinic appointment | 2249 | per service | NHS reference cost- EC services. 2022/2023 |  |
| Hospital physiotherapy appointment | 104 | per service | NHS reference cost- EC services. 2022/2023 |  |
| Hospital occupational therapy appointment | 206 | per service | NHS reference cost- EC services. 2022/2023 |  |
| Hospital speech and language therapy | 342 | per service | NHS reference cost- EC services. 2022/2023 |  |
| PD's nurse specialist at hospital | 58 | per service | NHS reference cost- EC services. 2022/2023 |  |
| Multi-disciplinary team (MDT) appointment with a group of health care professionals | 211 | per service | NHS reference cost- EC services. 2022/2023 |  |
| Hospital day case for a test or procedure (no overnight stay) not captured above | 1111 | per service | NHS reference cost- EC services. 2022/2023 |  |
| Ramps installed | 362 | Per unit | [Living Made Easy - Home](https://livingmadeeasy.org.uk/).  Accessed: December, 2024. |  |
| Rails, grab-rails | 6 | Per unit | [Living Made Easy - Home](https://livingmadeeasy.org.uk/).  Accessed: December, 2024. |  |
| Furniture raisers | 73 | Per unit | [Living Made Easy - Home](https://livingmadeeasy.org.uk/).  Accessed: December, 2024. |  |
| Stair-lift | 4500 | Per unit | [Living Made Easy - Home](https://livingmadeeasy.org.uk/).  Accessed: December, 2024. |  |
| State funded help: household activities | 18 | Per service | PSSRU, 2023; PSSRU 2024. Pg 10. | A midpoint was used between the PSSRU 2023 and 2024, given that this is a replacement cost and is more inherently more sensitive to labour-market changes. |
| Privately funded help: household activities | 18 | Per service | PSSRU, 2023; PSSRU 2024. Pg 10. | A midpoint was used between the PSSRU 2023 and 2024, given that this is a replacement cost and is more inherently more sensitive to labour-market changes. |
| Unpaid help: household activities | 18 | Per service | PSSRU, 2023; PSSRU 2024. Pg 10. | A midpoint was used between the PSSRU 2023 and 2024, given that this is a replacement cost and is more inherently more sensitive to labour-market changes. |
| State funded help: personal care | 18 | Per service | PSSRU, 2023; PSSRU 2024. Pg 10. | A midpoint was used between the PSSRU 2023 and 2024, given that this is a replacement cost and is more inherently more sensitive to labour-market changes. |
| Privately funded help: personal care | 18 | Per service | PSSRU, 2023; PSSRU 2024. Pg 10. | A midpoint was used between the PSSRU 2023 and 2024, given that this is a replacement cost and is more inherently more sensitive to labour-market changes. |
| Unpaid help: personal care | 18 | Per service | PSSRU, 2023; PSSRU 2024. Pg 10. | A midpoint was used between the PSSRU 2023 and 2024, given that this is a replacement cost and is more inherently more sensitive to labour-market changes. |
| State funded help: practical support | 18 | Per service | PSSRU, 2023; PSSRU 2024. Pg 10. | A midpoint was used between the PSSRU 2023 and 2024, given that this is a replacement cost and is more inherently more sensitive to labour-market changes. |
| Privately funded help: practical support | 18 | Per service | PSSRU, 2023; PSSRU 2024. Pg 10. | A midpoint was used between the PSSRU 2023 and 2024, given that this is a replacement cost and is more inherently more sensitive to labour-market changes. |
| Unpaid help: practical support | 18 | Per service | PSSRU, 2023; PSSRU 2024. Pg 10. | A midpoint was used between the PSSRU 2023 and 2024, given that this is a replacement cost and is more inherently more sensitive to labour-market changes. |
